# Innovatives SARS-CoV-2-Krisenmanagement im öffentlichen Gesundheitswesen: Corona-Dashboard und Abwasserfrühwarnsystem am Beispiel Berchtesgadener Land

**DOI:** 10.1007/s00103-021-03425-7

**Published:** 2021-10-01

**Authors:** Katalyn Roßmann, Gerd Großmann, Dimitrios Frangoulidis, Rüttger Clasen, Manuel Münch, Manfred Hasenknopf, Christian Wurzbacher, Andreas Tiehm, Claudia Stange, Johannes Ho, Marion Woermann, Jörg E. Drewes

**Affiliations:** 1 VI-2, Medical Intelligence & Information (MI2), Kommando Sanitätsdienst der Bundeswehr, München, Deutschland; 2 Landratsamt Berchtesgadener Land, Bad Reichenhall, Deutschland; 3 Lehrstuhl für Siedlungswasserwirtschaft, Technische Universität München, Am Coulombwall 3, 85748 Garching, Deutschland; 4 TZW: DVGW-Technologiezentrum Wasser, Karlsruhe, Deutschland

**Keywords:** Abwassermonitoring, Krisenmanagement, SARS-CoV-2-/COVID-19-Pandemie, Dashboard, Public Health, Crisis management, SARS-CoV-2/COVID-19 pandemic, Wastewater surveillance, Dashboard, Public health

## Abstract

**Hintergrund:**

Eine infektiologische Krisensituation wie die SARS-CoV-2-Pandemie stellte die Verwaltungsstrukturen des öffentlichen Gesundheitsdienstes vor erhebliche Herausforderungen, die abhängig von der personellen und digitalen Ausstattung in einer unterschiedlichen Effizienz des Ausbruchsmanagements resultierten. Die Einbindung von innovativen Instrumenten der Pandemiebekämpfung, wie Clusternachverfolgung, Risikogruppentestungen oder wie z. B. die von der EU-Kommission empfohlene Einbindung des Abwassermonitorings, wurde dadurch maßgeblich erschwert.

**Ziel:**

In dieser Fallstudie im Berchtesgadener Land stellen wir die Einbindung eines flächendeckenden georeferenzierten Abwassermonitorings vor, das seit Nov. 2020 95 % der gesamten Bevölkerung erfasst.

**Methoden:**

Für eine flächendeckende Erfassung erfolgte die Probennahme an 2 Tagen pro Woche an 9 kommunalen Kläranlagen und zusätzlich direkt aus der Kanalisation an 3 Standorten. Die Abwasserproben wurden direkt mittels Zentrifugation zur Feststoffabtrennung aufbereitet und über eine digitale Droplet Polymerase-Kettenreaktion (PCR) 4 spezifische Gene von SARS-CoV‑2 erfasst und quantifiziert.

**Ergebnisse:**

Die Einbindung des georeferenzierten Abwassermonitorings war erfolgreich. Die Abwasserbefunde werden für jede Gemeinde mit den Inzidenzen pro 100.000 Einwohnern dargestellt. Änderungen im Infektionsgeschehen sind 10 Tage vor den offiziellen Fallzahlen mit einer Sensitivität von ca. 20 pro 100.000 Einwohner erkennbar.

**Diskussion:**

Die Integration dieser innovativen Ansätze in eine umfassende Lageführung mittels eines digitalen Dashboards und der Etablierung eines Frühwarnsystems anhand eines quantitativen Abwassermonitorings resultierte im Landkreis Berchtesgadener Land in einem sehr effizienten, proaktiven Krisenmanagement. Dieses kann als Blaupause für andere Kommunen in Deutschland dienen.

## Abstract (en)

**Background:**

The rise of an infectious disease crisis such as the SARS-CoV‑2 pandemic posed significant challenges for the administrative structures of the public health service, which resulted in varying levels of efficiency in outbreak management as a function of staffing and digital resources. This substantially impeded the integration of innovative pandemic outbreak management tools. Innovative crisis management, such as cluster tracking, risk group testing, georeferencing, or the integration of wastewater surveillance recommended by the EU Commission, was made significantly more difficult.

**Aim:**

In this case study in Berchtesgadener Land, we present the integration of an area-wide georeferenced wastewater surveillance system that captured 95% of the entire population since November 2020.

**Methodology:**

Sampling occurred twice a week at nine municipal wastewater treatment plants and directly from the main sewer at three locations. Samples were pre-treated by centrifugation and subsequently analyzed by digital droplet polymerase chain reaction (PCR) targeting four specific genes of SARS-CoV‑2.

**Results:**

The integration of an area-wide georeferenced wastewater surveillance system was successful. Wastewater occurrences are plotted for each municipality against cumulative infections over seven days per 100,000 inhabitants. Changes in the infection pattern in individual communities are noticeable ten days ahead of the official case numbers with a sensitivity of approximately 20 in 100,000 inhabitants.

**Discussion:**

The integration of this innovative approach to provide a comprehensive overview of the situation by employing a digital dashboard and the use of an early warning system via quantitative wastewater surveillance resulted in very efficient, proactive management, which might serve as a blueprint for other municipalities in Germany.

## Einleitung

Der Öffentliche Gesundheitsdienst (ÖGD) in Deutschland wurde durch die SARS-CoV-2-Pandemie bezüglich seiner grundlegenden Aufgaben an seine Leistungsgrenze gebracht. Dies betrifft die Infektionskettenaufklärung, Infektionsquellenidentifizierung sowie die Infektionsprävention, hier speziell u. a. die Indexfallermittlung, die Kontaktpersonennachverfolgung und das Quarantänemanagement von Infizierten und Verdachtsfällen. In der Folge kam es zu Kapazitätsengpässen bei vielen Gesundheitsämtern [1]. Durch die Pandemie wurde offenbar, dass die etablierten Verwaltungsstrukturen der Gesundheitsämter, aber auch der anderen Einrichtungen des ÖGD in der Regel nicht vorbereitet sind, die Herausforderungen einer solchen infektiologischen Gefahrengroßlage bzw. dauerhaften Krisensituation unverzüglich, fehlerfrei, durchhaltefähig und in einer einheitlichen, kooperativen Führung zu bewältigen. Es zeigte sich eine sehr unterschiedliche Effektivität der Ämter gerade in der Kontaktnachverfolgung, die jeweils vom Ausmaß des lokalen Infektionsgeschehens, der personellen und digitalen Ausstattung sowie des bisher etablierten Ausbruchsmanagements abhing.

Viele Gesundheitsämter mussten verstärkt eigenverantwortlich handeln und passten ihre Strategien an. Primär wurde der Fokus auf eine effizientere Kontaktnachverfolgung gelegt, um eine Eindämmung der Infektionen zu erreichen. Nicht immer gelang es aber, die infektionsverursachenden Kontakte der Indexfälle zu ermitteln (Quellenidentifikation). Besonders problematisch war dabei der sehr unterschiedliche Grad der Digitalisierung der Meldesysteme in den Gesundheitsbehörden, der sich auf die Effizienz der Abläufe stark auswirkte. Ergänzende neue Herausforderungen der COVID-19-Pandemie waren und sind u. a. der zeitweise Mangel an diagnostischen Nachweissystemen, intensivmedizinischen und sonstigen Behandlungskapazitäten, Schutzausstattungen und Präventionsmöglichkeiten, wie Impfungen. In vielen Fällen wurde auf Verfahren und Protokolle aus dem Bereich der Katastrophenbekämpfung zurückgegriffen, da viele Elemente von dort auch für ein effektives und wirksames Infektionsausbruchsmanagement notwendig sind.

Je nach Altersgruppe, Grunderkrankungen und anderen Co-Faktoren treten asymptomatische bzw. mild bis moderate COVID-19-Infektionsverläufe unterschiedlich häufig auf. Es kommt zu einer hohen Anzahl an nicht erkannten Infektionen (sog. Dunkelziffer), die aber für den Verlauf des pandemischen Infektionsgeschehens von entscheidender Bedeutung ist. Neben dem Versuch, diese Fälle durch eine Strategie mit flächendeckenden, z. T. anlasslosen, jederzeit verfügbaren Testangeboten zu erkennen, hat sich eine andere Methodik als sinnvoll und nützlich herausgestellt, die für die betroffenen, infizierten Personen weniger invasiv ist – das infektiologische Abwassermonitoring als Teilgebiet der Abwasserepidemiologie.

Die Abwasserepidemiologie (engl.: „wastewater-based epidemiology“, WBE) bekommt in den Umweltwissenschaften zunehmend Zuspruch als eine diagnostische Methode, um den Konsum von Drogen und Medikamenten für gesamte Siedlungsgebiete abzuschätzen [2]. Die Methodik eignet sich aber auch dazu, die Verbreitung von Infektionserregern und deren genotypische Eigenschaften (z. B. Resistenzgene, sonstige Mutationen) zu erfassen. So scheiden Infizierte SARS-CoV‑2 nicht nur über den Speichel, sondern auch über den Magen-Darm-Trakt mit dem Stuhl aus. Im Urin ist das Virus in der Regel nicht nachweisbar ([3, 4]; Tab. 1).

**Table 1:** 

Referenz	Land	Analysemethode	Art der Probe	Anzahl an positiv getesteten Personen/Gesamtzahl an getesteten Personen (Anteil in %); [zusätzliche Information]
*Cai et al. 2020* 10.1093/cid/ciaa198	China	RT-PCR	Stuhl	5/6 (83 %)
*Chan et al. 2020* 10.1016/S0140-6736(20)30154-9	China	RT-PCR	Stuhl	0/7 (0 %)
*Chen et al. 2020* 10.1080/22221751.2020.1732837	China	RT-PCR	Analabstriche	11/28 (39 %)
*Chen et al. 2020* 10.7326/M20-0991	China	RT-PCR	Stuhl	12/19 (63 %)
*Holshue et al. 2020* 10.1056/NEJMoa2001191	USA	RT-PCR	Stuhl	1/1 (100 %)
*Kujawski et al. 2020* 10.1101/2020.03.09.20032896	USA	RT-PCR	Stuhl	7/10 (70 %)
*Ling et al. 2020* 10.1097/CM9.0000000000000774	China	RT-PCR	Stuhl Urin	54/66 (82 %) 4/58 (7 %)
*Lescure et al., 2020* 10.1016/S1473-3099(20)30200-0	Frankreich	RT-PCR	Stuhl Urin	2/5 (40 %) 0/5 (0 %)
*Lo et al. 2020* 10.7150/ijbs.45357	China	RT-PCR	Stuhl Urin	10/10 (100 %) 0/10 (0 %)
*Pan et al. 2020* 10.1016/S1473-3099(20)30113-4	China	RT-qPCR	Stuhl	9/17 (53 %) [Zwischen Tag 0–11 konnten 550‑1,21 × 10^5^ Genkopien nachgewiesen werden]
*Tang et al. 2020* 10.3201/eid2606.200301	China	RT-PCR	Stuhl	1/3 (33 %)
*Wu et al. 2020* 10.1016/S2468-1253(20)30083-2	China	RT-PCR	Stuhl	41/74 (55 %) [Patient wurde nach 47 Tagen noch positiv getestet, durchschnittlich waren Kotproben 11 Tage länger positiv als respiratorische Proben]
*Wölfel et al. 2020* 10.1038/s41586-020-2196-x	Deutschland	RT-qPCR	Stuhl	4/4 (100 %)
		Zellkultur	–	0/4 (0 %)
		RT-qPCR	Urin	0/9 (0 %)
*Wang et al. 2020* 10.1001/jama.2020.3786	China	RT-PCR	Stuhl	44/153 (29 %)
		Zellkultur von 4 Proben mit hohen Genomzahlen		Es konnten kultivierbare Viren nachgewiesen werden
*Xiao et al. 2020* 10.1053/j.gastro.2020.02.055	China	RT-PCR	Stuhl	39/73 (43 %)
*Xiao et al. 2020* 10.3201/eid2608.200681	China	RT-qPCR	Stuhl	12/28 (43 %)
		Zellkultur		2/3 (33 %) [In 2 von 3 untersuchten Proben konnten vermehrungsfähige Viren nachgewiesen werden]
*Xu et al. 2020* 10.1038/s41591-020-0817-4	China	RT-PCR	Stuhl	8/10 (80 %)
*Young et al. 2020* 10.1001/jama.2020.3204	Singapur	RT-PCR	Stuhl	4/8 (50 %)
*Zhang et al. 2020* 10.1002/jmv.25742	China	RT-PCR	Stuhl	8/22 (36 %)
*Zhang et al. 2020* 10.1080/22221751.2020.1729071	China	RT-PCR	Analabstriche	14/31 (45 %)
*Zhang et al. 2020* 10.46234/ccdcw2020.033	China	Zellkultur und Elektronenmikroskopie	Stuhl	1/1 (100 %) [Nachweis von vermehrungsfähigen Viren]
*Zhou et al. 2020* 10.1038/s41591-020-0912-6	China	Zellkultur	Stuhl	1/1 (100 %) [Nachweis von vermehrungsfähigen Viren]
*Sharma et al. 2021* 10.1371/journal.pone.0253355	Indien	RT-PCR	Urin	0/130 (0 %)
*Peng et al. 2020* 10.1002/jmv.25936	China	RT-PCR	Urin	1/1 (100 %)
*Shirazi et al. 2021* 10.3390/jcm10061158	Länderübergreifende Studien	RT-PCR	Speichel	>80 %
*Comber et al. 2020* 10.1002/rmv.2185				
*Mohammadi et al. 2020* 10.1016/j.ebiom.2020.102903				
*Lippi et al. 2020,* 10.23750/abm.v91i3.10187				

*RT-PCR* Reverse-Transkriptase-Polymerase-Kettenreaktion (englisch: „reverse transcription polymerase chain reaction“, die RT-PCR ist eine Nachweismethode für spezifische Ribonukleinsäuren, die auf der Synthese und Vervielfältigung von komplementärer DNA beruht), *RT-qPCR* Reverse-Transkriptase-quantitative-Polymerase-Kettenreaktion („reverse transcription quantitative polymerase chain reaction“, Weiterentwicklung der RT-PCR, die auch eine Quantifizierung der Ausgangs-RNA ermöglicht)

Mit der Abwasserepidemiologie ist es möglich – unabhängig von einer individuellen Bereitschaft, an klinischen Teststrategien teilzunehmen –, das Ausmaß des Infektionsgeschehens für ein gesamtes Siedlungsgebiet über SARS-CoV-2-Biomarker im Abwasser objektiv und durchgehend zu erfassen [5]. Der Nachweis von SARS-CoV-2-Biomarkern im Abwasser basiert auf quantitativen Reverse-Transkriptase-Polymerase-Kettenreaktion-(RT-PCR-)Tests, die von Forschergruppen in Deutschland, Frankreich, den Niederlanden, China und den USA veröffentlicht wurden [6–10]. Am 17.03.2021 empfahl die EU-Kommission den Mitgliedsstaaten die Einführung eines flächendeckenden SARS-CoV-2-Abwassermonitorings als ein weiteres ergänzendes diagnostisches Instrument der Pandemiebekämpfung [11].

Aufgrund rapide steigender 7‑Tage-Inzidenzen (Anstieg der gemeldeten SARS-CoV-2-Fälle von 3,8/100.000 Einwohner am 06.10.2020 auf 252/100.000 Einwohner am 19.10.2020) war das Landratsamt Berchtesgadener Land in Abstimmung mit der Bayerischen Staatsregierung und der Regierung von Oberbayern gezwungen, umfassende Beschränkungen im Rahmen einer Allgemeinverfügung festzulegen, um das Infektionsgeschehen zu bremsen. Damit befand sich das Berchtesgadener Land faktisch als erster Landkreis in Deutschland im Zuge der 2. Pandemiewelle in einem Lockdown, der durch den anschließend von Bund und Freistaat Bayern verhängten Lockdown mit erheblichen Einschränkungen für das öffentliche Leben bis zum Juni 2021 anhielt.

**Figure 1:**
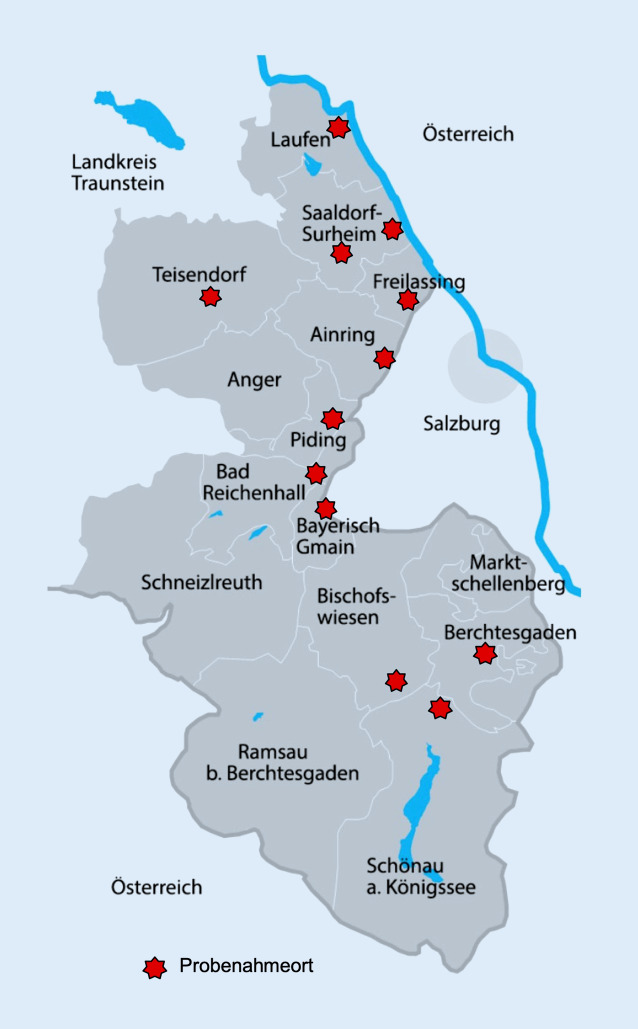

Das Berchtesgadener Land ist ein von Tourismus und Pendlern geprägter Landkreis mit rund 106.000 Einwohnern in der Grenzregion zu Österreich (Abb. 1). Im Rahmen der Corona-Amtshilfe erfolgte seit September 2020 eine Beratung durch Pandemieexperten der Bundeswehr zur Evaluierung und Anpassung des örtlichen Krisenmanagements. Ergänzt wurden die Maßnahmen des Krisenstabs ab November 2020 durch ein flächendeckendes SARS-CoV-2-Biomarker-Abwassermonitoring zur Früherkennung von Änderungen im lokalen Infektionsgeschehen sowie des Auftretens von Virusvarianten im Landkreis im Rahmen des vom Bundesministerium für Bildung und Forschung (BMBF) geförderten Forschungsvorhabens „Biomarker CoV2“.

Die Ergebnisse des Monitorings wurden gemeinsam mit den lokal diagnostizierten Infektionen und digitalen Geoinformationen zu kritischen Infrastrukturen (KRITIS) des Landkreises, wie zum Beispiel Abwasserentwässerungssysteme, Pflegeheime, Kitas und Schulen, in einem Dashboard zur Lageführung fusioniert. Die Visualisierung von Informationen mittels solcher, häufig durch Geoinformationssysteme (GIS) gestützter Dashboards hat sich seit Beginn der COVID-19-Krise zunehmend als weitverbreitetes Hilfsmittel etabliert [12]. Ziel war es, den Entscheidern des Krisenstabs vor Ort eine objektive Grundlage zur Beurteilung der weiteren Maßnahmen in der Pandemiebekämpfung im Landkreis zur Verfügung zu stellen. Gemäß der Empfehlung der EU-Kommission, das Abwassermonitoring flächendeckend einzusetzen [11], dient der Landkreis Berchtesgadener Land als bundesweites Musterbeispiel für eine effiziente Integration von Innovationen in das Krisenmanagement der Ämter.

Die vorliegende Arbeit dokumentiert zunächst die Einbindung der Abwasserbefunde für die Lagebeurteilung sowie die angewandte Messmethode und berichtet dann über die Anwendung des Abwassermonitorings im Landkreis Berchtesgadener Land und ausgewählte Ergebnisse.

## Methoden

### Evaluierung des Ausbruchsmanagements und der Corona-Dashboard-Entwicklung

Im Rahmen des Krisenmanagements wurde im Oktober 2020 ein Modell zur Entwicklung eines möglichst alle krisenrelevanten Aspekte umfassenden Corona-Dashboards (longitudinaler Ansatz) für den lokalen Krisenstab des Landratsamtes Berchtesgadener Land auf Basis des Geoinformationssystems ArcGIS^1^ mit einem Schwerpunkt in der Etablierung eines Abwassermonitoringfrühwarnsystems zunächst im Landratsamt vorgestellt. Ziel war es, durch die Verschneidung der Messwerte des Abwassermonitorings mit den Geoinformationen der lokalen Behörden ein aktuelles Lagebild zur Viruslast im Abwasser zu visualisieren, Rückschlüsse auf örtliche Schwerpunkte des Viruseintrags in die Kanalisation zu ziehen und schließlich basierend auf diesen Erkenntnissen mit gezielten Maßnahmen die Verbreitung der Infektion zu unterbrechen.

Die Umsetzung wurde seitens der Bundeswehrexperten und der Wissenschaftler der Technischen Universität München sowie des Technologiezentrums Wasser (TZW) Karlsruhe fachlich und methodisch unterstützt. Das Agendasetting und die Implementierung wurden ressortübergreifend im vernetzten Ansatz betrieben, unter Beteiligung des Landrats- und des Gesundheitsamts sowie der Bürgermeister aller Gemeinden und zahlreicher zusätzlicher Experten [13].

### Bestimmung von SARS-CoV-2-Biomarkern in Abwasserproben und Datenauswertung

In dieser Studie erfolgte die wöchentliche Abwasserprobenahme in den einzelnen Kommunen des Landkreises jeweils am Montag- und Mittwochmorgen als qualifizierte Stichprobe mit automatischen Probennehmern, was einen Probenversand am Nachmittag des gleichen Tages und einen zügigen Rücklauf der Befunde sicherstellte. Die Abwasserprobenahme erfolgte je nach Standort in einem Zeitintervall von 4–6 h von den frühen Morgenstunden bis Mittag in den einzelnen Kommunen des Landkreises. So konnten Befunde der Abwasserproben mit einem Verzug von weniger als 48 h jeweils Mittwoch bzw. Freitag an den Krisenstab gemeldet werden.

Um die Bevölkerung im Landkreis Berchtesgadener Land möglichst flächendeckend zu erfassen, erfolgte die Probennahme für SARS-CoV-2-Biomarker an 9 kommunalen Kläranlagen (mit je ca. 1300 bis 21.000 angeschlossenen Einwohnern) und zusätzlich direkt aus der Kanalisation an 3 Probenahmestellen (Hauptsammler; Abb. 1).

Unter Berücksichtigung der angeschlossenen Einwohner wurden durch diese Probennahme ca. 100.700 Einwohner im Landkreis Berchtesgadener Land erfasst, das entspricht 95 % der Gesamtbevölkerung. Die Verweilzeit des Kommunalabwassers bis zu den Probenahmeorten betrug i. d. R. weniger als 2 h. Die Probenahme erfolgte durch das Klärwerkspersonal der beteiligten Kommunen. Die Proben wurden gekühlt und am gleichen Tag via Expresszustellung an das Labor des TZW in Karlsruhe geschickt. Zusätzlich zum Kommunalabwasser erfasst die Kanalisation je nach Zustand und Alter sogenanntes Fremdwasser, das in die Kanalisation einsickert und zu einer Verdünnung des Kommunalabwassers führt. Diese Anteile variierten bei den beteiligten Kommunen zwischen 0 % und 70 % und wurden bei der Kalkulation der Biomarkerkonzentrationen als Verdünnungsfaktor berücksichtigt, um eine bessere Vergleichbarkeit der Befunde an den einzelnen Messstellen zu gewährleisten.

Um die Populationsdynamik in den SARS-CoV-2-Daten, vor allem aber auch weitere Verdünnungseffekte im Kommunalabwasser durch Regen- und Tauwetterereignisse am Tag der Probenahme abzubilden, kann eine Normalisierung der gemessenen Viruslast zu anderen Viren, die im menschlichen Darmtrakt allgegenwärtig sind (wie z. B. CrAss-Bakteriophagen), in Betracht gezogen werden. CrAss-Phage ist ein Virus, das Bakterien infiziert (Bakteriophage) und sehr spezifisch und in hoher Konzentration in menschlichem Fäkalienmaterial auftritt [14]. CrAss-Phagen wurden auch in dieser Studie erfasst und als interner Surrogatparameter genutzt.

**Figure 2:**
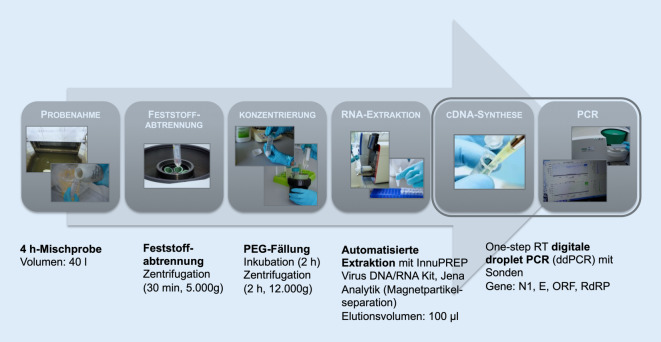

Die Abwasserproben (je 40 ml) wurden direkt nach dem Eintreffen im Labor mittels Zentrifugation zur Feststoffabtrennung aufbereitet (Abb. 2). Die Viren im Überstand wurden über eine Polyethylenglykolfällung aufkonzentriert und die viralen Nukleinsäuren in einer automatisierten Extraktion (innuPREP Virus DNA/RNA Kit und innuPure C16 System) über magnetische Beads (magnetische Nano- oder Mikropartikel) isoliert und aufgereinigt [15].

Über eine Digitale Droplet PCR (ddPCR) wurden folgende spezifische Gene von SARS-CoV‑2 erfasst und quantifiziert: Nukleokapsid-Gen N, Hüllprotein-Gen E, Replikase-Polyprotein-(Open-Reading-Frame‑)Gen ORF und RNA-abhängiges RNA-Polymerase-Gen RdRP. Ein positiver Befund wurde ermittelt, wenn mindestens 2 Gene bei der PCR-Analytik detektiert wurden. Das N1-Gen zeigte zwar eine hohe Sensitivität, aber auch hohe willkürliche Schwankungen und wurde daher von der Auswertung ausgeschlossen. Für die Auswertung in dieser Studie wurden das Gen E sowie der Durchschnitt aus allen 3 Zielgenen (E, ORF, RdRP) als Genkopien pro ml Abwasser (Abundanzen) dargestellt. Basierend auf dem Ausgangsvolumen an Abwasser und dem Volumen an RNA-Extrakt, das in der PCR-Reaktion eingesetzt wird, konnte eine Nachweisgrenze von 2,5 Genkopien pro ml Abwasser errechnet werden.

Die Co-Quantifizierung von viralen Nukleinsäuren aus CrAss-Phage wurde in die SARS-CoV-2-Abwasserüberwachung integriert, um die Variabilität der SARS-CoV-2-Biomarker-Ergebnisse durch die unterschiedliche Verdünnung der menschlichen Fäkalien, z. B. bei Regenereignissen, zu erfassen. Außerdem kann der Nachweis von CrAss-Phage als Prozesskontrolle für die Anreicherung und Aufkonzentrierung genutzt werden. Die Bestimmung von spezifischen DNA-Sequenzen für CrAss-Phage erfolgte mittels quantitativer Realtime PCR mit SYBR Green [14, 15].

## Ergebnisse

### Anpassung des Pandemiemanagements im Berchtesgadener Land

**Figure 3:**
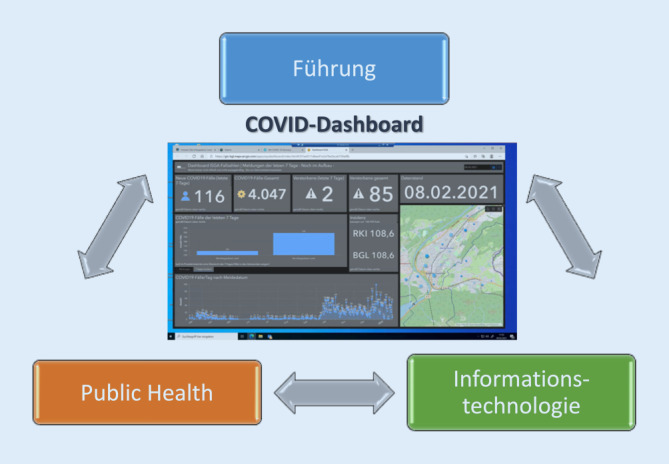

Um die Verwaltungsstrukturen dem pandemischen, aus der Routine gebrachten Kontext anzupassen, wurde im Berchtesgadener Land ein alternatives Krisenmanagement anhand eines aufgrund der Erfahrungen aus der Ebolakrise in Westafrika entwickelten Modells adaptiert und erweitert. Dieses Modell enthielt bereits ab Herbst 2020 alle notwendigen thematischen Bereiche derzeit aktueller Strategien [16–19]. Das Modell zeichnet sich insgesamt durch 3 tragende Elemente aus: Führung – Public Health – Informationstechnologie (Abb. 3).

Dabei bündelt das Element der *Führung* das für die Krisenbewältigung notwendige Wissensmanagement, die Ressourcensteuerung (Personal, Ausbildung, Infrastruktur, Material/IT, Konzepte, Kooperationen), die Risikokommunikation (Allgemeinheit, med. Fachpersonal, Presse, soziale Medien, Radio, Anti-Fake-News), die Evaluierung des Ausbruchsmanagements, Studien (Forschung u. Entwicklung) sowie die innere Führung (u. a. Mitarbeitermotivation, Gruppenzusammenhalt). Das fachliche Management (*Public Health*) beinhaltet die Epidemiologie mit den beiden Kernelementen der Investigation (Kontaktnachverfolgung/Infektkettenaufklärung) und Surveillance (Abwassermonitoring), Testen, Diagnostik, Patientenversorgung (ambulant, prä-/klinisch) und Therapie, Vollzug, Impfen, Gesundheitsförderung/-vorsorge sowie psychosoziale Unterstützung Betroffener und Beteiligter. Das dritte Element nutzt die *Digitalisierung* durch eine vernetzte, standardisierte Software für ein besseres Daten‑, Informations- und Wissensmanagement, Prozessoptimierung sowie die Krisen- und Strukturanpassung. Die Digitalisierung mündete in der Bündelung unterschiedlicher Datenströme in übersichtlichen Dashboards. Im Landkreis Berchtesgadener Land wurden dafür folgende Themen priorisiert:

- Gesamtübersicht zum *Verlauf des Infektionsgeschehens* auf Gemeindeebene,
- Testen anhand der *Anzahl durchgeführter Tests und Testergebnisse* aus den kommunalen Testzentren und weiteren Einrichtungen,
- *Surveillance anhand von Abwassermonitoring* als Frühwarnsystem; gleichzeitig ist das kommunale digitale Abwasserkataster hinterlegt, das in den Siedlungsgebieten auch eine gezielte höher aufgelöste Abwasserbeprobung von einzelnen Ortsteilen oder Straßenzügen zulässt,
- Investigation: *Clusteranalyse anhand georeferenzierter Index- und Kontaktpersonen*^2^,
- *Monitoring Vollzug in kritischen Einrichtungen* gem. §§ Infektionsschutzgesetz (IfSG): Übersicht über 65 Pflegeeinrichtungen, Krankenhäuser etc. mit Kontrolle des Vollzugs und Umsetzung der Teststrategie i. R. des IfSG und Ausbruchsmanagements,
- Monitoring der Quarantäne bei Pendlern^3^,
- Patientenversorgung^4^,
- und *Impfen*^5^.

### Abwassermonitoring als ein diagnostisches Instrument

Zusätzlich zur Individualtestung kann ergänzend das Abwassermonitoring eingesetzt werden, welches mit wenigen Testungen über SARS-CoV-2-Ausscheidungen über den Stuhl Infektionen in der Gesamtbevölkerung des Einzugsgebiets erfassen kann. Die Befunde der Abwasserproben wurden mit einem Verzug von weniger als 48 h an den Krisenstab gemeldet.

**Figure 4:**
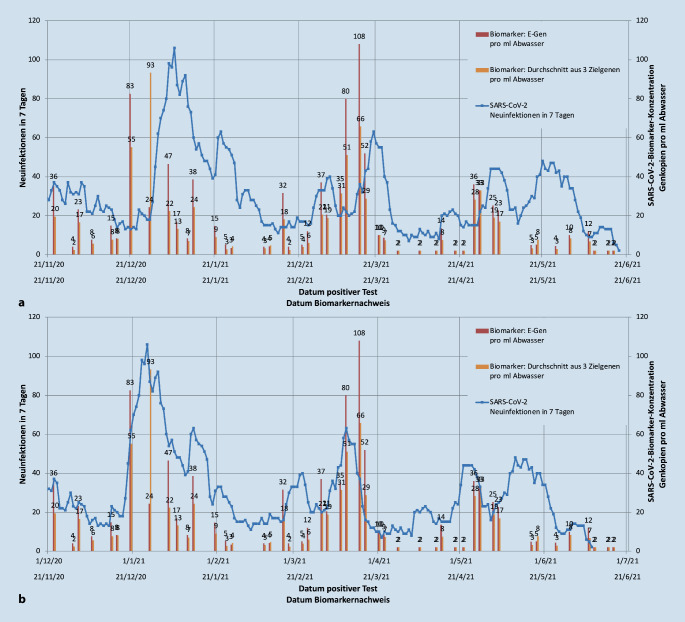

Die Ergebnisse wurden für jede Gemeinde fortlaufend über die Zeit mit den Neuinfektionen der letzten 7 Tage sowie Neuinfektionen pro Tag aufgetragen. Der Zusammenhang zwischen beobachteten Abundanzen im Abwasser und berichteten aggregierten Neuinfektionen in 7 Tagen ist in Abb. 4a beispielsweise für die Marktgemeinde Berchtesgaden mit den angeschlossenen Umlandgemeinden Bischofswiesen, Ramsau b. Berchtesgaden und Schönau a. Königssee im Zeitraum November 2020 bis Juni 2021 illustriert. Das Infektionsgeschehen ist in diesem Zeitraum sehr dynamisch, mit einem deutlichen Rückgang der Fallzahlen in der zweiten Novemberhälfte und einem starken Wiederanstieg Ende Dezember/Anfang Januar, einem Abflachen im Februar mit erneutem Anstieg Anfang März. Der Versatz der Biomarkerbefunde um 10 Tage nach vorn zeigt eine Passung mit der Kurve der nachgewiesenen Neuinfektionen (Abb. 4b).

### Pandemiemanagement im Berchtesgadener Land

**Figure 5:**
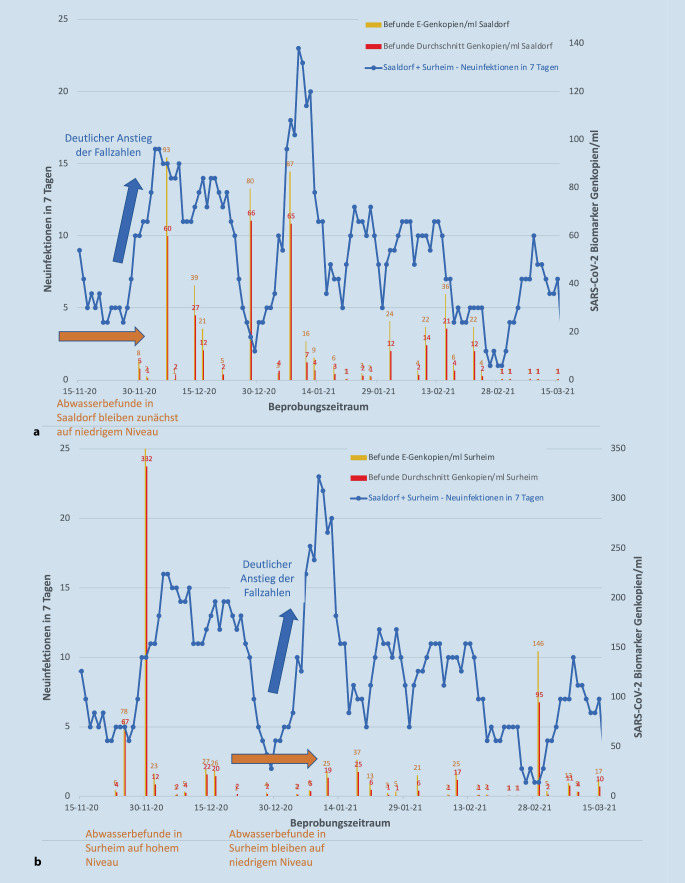

Aufgrund des Vorlaufs des Abwassermonitorings wurden bei deutlich höheren Abwasserbefunden wiederholt gezielte präventive Maßnahmen veranlasst, die zur Eingrenzung von Clustern in einzelnen Kommunen führte. Cluster von Infizierten lassen sich so auch lokal verorten. Exemplarisch ist dies für die Gemeinde Saaldorf-Surheim illustriert (Abb. 5). Die Gemeinde besteht aus 2 Ortsteilen mit jeweils eigenen Kläranlagen (mit je ca. 1300 bzw. 2900 angeschlossenen Einwohnern). Die Infektionszahlen werden jedoch für beide Ortsteile aggregiert berichtet.

Der starke Anstieg des Infektionsgeschehens Anfang Dezember 2020 deutete sich im Ortsteil Surheim durch deutlich erhöhte Abundanzen bereits einige Tage vorher an, während die Abundanzen im Ortsteil Saaldorf erst einige Tage später anstiegen. Der deutliche Anstieg der Infektionen Anfang Januar 2021 war aufgrund der signifikant erhöhten Abundanzen im Abwasser eher durch den Ortsteil Saaldorf dominiert, während die Abundanzen in Surheim auf niedrigem Niveau blieben. Der deutliche Wiederanstieg der Infektionen Anfang März 2021 war dagegen durch das Infektionsgeschehen im Ortsteil Surheim geprägt, ohne Auffälligkeiten im Ortsteil Saaldorf.

## Diskussion

### Anpassung des Pandemiemanagements im Berchtesgadener Land – Lernen aus der Krise

Bei den Strategien im Berchtesgadener Land wurden im Wesentlichen die Bereiche Testen, Kontaktnachverfolgung, Einreisemanagement (Pendlerverkehr und Vollzug), Impfungen, Hygiene/-regeln, Forschung, Surveillance, Risikokommunikation berücksichtigt. Eine umfassende Digitalisierung im Sinne eines zentralen digitalen und alle Aspekte umfassenden Dashboards zur übersichtlichen und effektiven Lageführung (Abb. 3) ist bis dato in der Umsetzung nicht bekannt – weder auf kommunaler, noch regionaler, Landes‑/Bundes- oder internationaler Ebene. Zwar gibt es zahlreiche einzelne Dashboards zu verschiedenen dieser Themen, es fehlt aber an vollumfänglichen Versionen. Weiterhin wenig im Fokus der Landratsämter sind Gesundheitsfürsorge/-vorsorgeprogramme für COVID-19-Risikogruppen sowie die psychosoziale Unterstützung und Betreuung von Betroffenen (SARS-CoV-2-Positiven sowie COVID-19-Patienten) und anderer Beteiligter (z. B. Fachpersonal sowie trainierte Laien in Pflege und Gesundheitsämtern).

Die Kontaktnachverfolgung wurde im Berchtesgadener Land zunächst über ein eigens dafür konzipiertes Microsoft-Excel™-basiertes System durchgeführt, das dann Anfang Dezember 2020 durch die bayerische Software zur Kontaktnachverfolgung BaySIM [20] und in weiterer Folge Anfang April 2021 durch SORMAS [21] abgelöst wurde. Das „Surveillance Outbreak Response Management and Analysis System“ (Version SORMAS X) wird aktuell durch ein Konsortium (Akademie für Öffentliches Gesundheitswesen Düsseldorf, netzlink, Helmholtz-Zentrum für Infektionsforschung, vitagroup) betrieben und mit Schnittstellen für die Systeme SurvNet und DEMIS des Robert Koch-Instituts ausgestattet. Mitte August 2021 war es bei 347 Gesundheitsämtern in Deutschland installiert. In Bayern nutzen derzeit ca. knapp 20 der insgesamt 76 Gesundheitsämter das System. Es liefert Kontaktübersichten, Überwachungsübersichten und kann Infektionsketten darstellen.

Sobald die Schnittstellenfähigkeiten etabliert sind, sind die Verknüpfungen mit dem ArcGIS-System im Berchtesgadener Land, das die Grundlage des Dashboards darstellt, bereits zwischen den jeweils zuständigen Organisationen bzw. Unternehmen vorbereitet, da auch SORMAS X bei Weitem nicht alle notwendigen Aspekte eines vollumfänglichen Dashboards ermöglicht. Vielmehr könnte es einen wichtigen Beitrag als einheitlicher Datenlieferant (Contributors) mit dem Teillagebild „Infektionsnachverfolgung“ für die Gesamtlage liefern. Ziel eines solchen Dashboards ist es auch, dass die benötigten Zusammenfassungen nicht erst in Einzelarbeit in den meldenden Abteilungen erstellt werden müssen, sondern automatisiert aufbereitet und eingelesen werden können. Dies bietet eine spürbare Entlastung des dafür eingesetzten Personals hinsichtlich zeitaufwendiger Fleißarbeit und damit Freiwerden von Kapazitäten für eine intensivere Analyseleistung.

### Abwassermonitoring als ein innovatives diagnostisches Instrument

Trotz einer nur zweimaligen Probenahme pro Woche bilden die gemessenen Abundanzen die Änderungen im Infektionsgeschehen mit einem Vorlauf von ca. 10 Tagen vor den offiziellen Fallzahlen sehr gut ab (Abb. 4). Weiterhin ergeben sich in der Darstellung der Neuinfektionen in 7 Tagen klare Muster in den gemessenen Abundanzen, die es zulassen, sowohl einen deutlichen Anstieg der Infektionen als auch einen Rückgang des Infektionsgeschehens zu prognostizieren. Gerade bei Trends mit fallenden Fallzahlen ist auffällig, dass die Abundanzen sehr schnell abnehmen, was nahelegt, dass die Virenlast in den Ausscheidungen von Infizierten nach überstandener Infektion rasch abnimmt, was Erfahrungen aus internationalen und nationalen Studien bestätigt [22–24].

Die Auswertung in den Einzugsgebieten zeigt, dass Fallzahlen im Bereich von ca. 20 Infizierten pro 100.000 Einwohnern zu einem positiven Signal bei Nachweis von mindestens 2 spezifischen SARS-CoV-2-Sequenzen führten. Es ist jedoch zu beachten, dass ein Probenahmefenster von nur 4 h in den frühen Morgenstunden möglicherweise nicht ausreicht, die Ausscheidungen von allen Infizierten zu erfassen.

### Innovatives Pandemiemanagement im Berchtesgadener Land

Die durch das Abwassermonitoring ermittelten Muster (exemplarisch für die Gemeinde Saaldorf-Surheim, Abb. 5) zeigen daher eine Änderung des Infektionsgeschehens einige Tage vor Anstieg der gemeldeten Neuinfektionen deutlich an. Am Beispiel der Gemeinde Saaldorf-Surheim lässt sich anschaulich aufzeigen, dass das Abwassermonitoring nicht nur als „Frühwarnsystem“, sondern auch als „Entwarnsystem“ dienen kann.

Durch den deutlichen Anstieg der Abundanzen in Surheim Anfang März legte der Krisenstab im Landratsamt den Fokus auf die nochmalige und gezielte Analyse der Infektionsketten in Surheim. Die Infektionsherde waren zu diesem Zeitpunkt noch klar eingrenzbar. Folglich konnten durch die intensiven Bemühungen der Contact-Tracing-Team-Mitarbeiter noch weitere Kontaktpersonen ermittelt und in häusliche Quarantäne versetzt werden. Viele dieser bereits in Quarantäne befindlichen Kontaktpersonen entwickelten einige Tage später ebenfalls Symptome und wurden positiv auf COVID-19 getestet. Die Infektionsketten wurden mithilfe der Ergebnisse aus dem Abwasserscreening somit frühzeitig unterbrochen und dadurch ein diffuses Infektionsgeschehen verhindert. Durch die im gleichen Zeitraum anhaltend niedrigen Abundanzen in Saaldorf, konnte für diesen Ortsteil „Entwarnung“ gegeben werden, sodass sich die Bemühungen des Gesundheitsamtes voll auf die erfolgreiche Analyse der Infektionsketten in Surheim fokussieren konnten.

## Fazit

Am Beispiel des Berchtesgadener Landes zeigt sich, dass innovatives Krisenmanagement in der Public-Health-Krise nötig und möglich ist. Die Integration dieser innovativen Ansätze einer umfassenden Lageführung anhand eines digitalen Dashboards und der Etablierung eines Früh- und Entwarnsystems anhand eines quantitativen Abwassermonitorings resultierte im Landkreis Berchtesgadener Land in einem sehr effizienten, proaktiven Krisenmanagement, das als Vorlage für andere Kommunen – aber letztlich auch auf Länder- sowie auf Bundesebene im jeweils notwendigen Detaillierungsgrad – dienen kann. Die entwickelten Dashboards können auch bei anderen, zukünftigen Infektionsgeschehen eingesetzt werden. Eine Limitierung in der flächendeckenden Implementierung dieses Frühwarnsystems liegt neben der wünschenswerten, aber herausfordernden Standardisierung der Datenübertragung bei der Harmonisierung der analytischen Verfahren. Hier gilt es, den zuständigen und daran interessierten Kommunen zeitnah innovative Lösungsansätze anzubieten. Eine rechtliche Verankerung der Abwasser-Surveillance als kosteneffiziente wie flächendeckende Ergänzung zum individuellen Testen wäre aus einer Public-Health-Perspektive sinnvoll. Hierzu hat sich die EU-Kommission inzwischen entsprechend positioniert [11].

## Footnotes

1 https://www.esri.com/de-de/arcgis/products/arcgis-dashboards/overview.

2 Für die Aufbereitung weiterer Statistiken aus SORMAS X zur Kontaktnachverfolgung wird derzeit die Schnittstellenmodulation abgewartet.

3 Aktuell wird eine Schnittstelle zur digitalen Einreiseanmeldung DEA entwickelt (https://www.einreiseanmeldung.de/#/).

4 Automatische Schnittstelle zum DIVI-Register des Robert Koch-Instituts ist funktionsfähig.

5 Derzeit sind die Schnittstellen aufgrund des Datenschutzes noch nicht moduliert.
